# Non-metabolic enzyme function of pyruvate kinase M2 in breast cancer

**DOI:** 10.3389/fonc.2024.1450325

**Published:** 2024-10-01

**Authors:** Mohammed Jemal, Mamaru Getinet, Gashaw Azanaw Amare, Bantayehu Addis Tegegne, Temesgen Baylie, Enyew Fenta Mengistu, Enatnesh Essa Osman, Nuredin Chura Waritu, Adane Adugna

**Affiliations:** ^1^ Department of Biomedical Science, School of Medicine, Debre Markos University, Debre Markos, Ethiopia; ^2^ Department of Medical Laboratory Sciences, College of Health Sciences, Debre Markos University, Debre Markos, Ethiopia; ^3^ Department of Pharmacy, College of Medicine and Health Sciences, Debre Markos University, Debre Markos, Ethiopia; ^4^ Department of Biomedical Sciences, School of Medicine, Wolaita Sodo University, Wolaita Sodo, Ethiopia

**Keywords:** breast cancer, non-metabolic enzyme function, pyruvate kinase M2, noncoding RNA, tumorigenesis

## Abstract

Breast cancer (BC) is a prevalent malignant tumor in women, and its incidence has been steadily increasing in recent years. Compared with other types of cancer, it has the highest mortality and morbidity rates in women. So, it is crucial to investigate the underlying mechanisms of BC development and identify specific therapeutic targets. Pyruvate kinase M2 (PKM2), an important metabolic enzyme in glycolysis, has been found to be highly expressed in BC. It can also move to the nucleus and interact with various transcription factors and proteins, including hypoxia-inducible factor-1α (HIF-1α), signal transducer and activator of transcription 3 (STAT3), β-catenin, cellular-myelocytomatosis oncogene (c-Myc), nuclear factor kappa-light-chain enhancer of activated B cells (NF-κB), and mammalian sterile 20-like kinase 1 (MST1). This interaction leads to non-metabolic functions that control the cell cycle, proliferation, apoptosis, migration, invasion, angiogenesis, and tumor microenvironment in BC. This review provides an overview of the latest advancements in understanding the interactions between PKM2 and different transcription factors and proteins that influence the initiation and progression of BC. It also examined how natural drugs and noncoding RNAs affect various biological processes in BC cells through the regulation of the non-metabolic enzyme functions of PKM2. The findings provide valuable insights for improving the prognosis and developing targeted therapies for BC in the coming years.

## Introduction

Breast cancer is the most commonly occurring cancer in women and the most common cancer overall (1). There were more than 2.26 million new cases of BC, and approximately 685 000 women died from the disease in 2020 (2). It can be divided into three main subtypes: luminal, HER2-positive, and triple-negative breast cancer (TNBC) (3). TNBC, in particular, is known for its high invasiveness. Around 30% of individuals with early-stage BC experience metastases, resulting in a 5-year relative survival rate of 25% (4). Nonetheless, the precise molecular mechanisms responsible for BC development across different subtypes remain unclear. Further exploration is needed to identify specific biomarkers that can be targeted to improve the overall prognosis of patients with this disease (5).

The primary distinguishing feature of cancer cells is their metabolic reprogramming. Unlike healthy cells, tumor cells rely on aerobic glycolysis for energy production, even when enough oxygen present in the environment (6, 7). This shift from the typical respiratory pathway to aerobic glycolysis is referred to as the Warburg effect (7, 8). Aerobic glycolysis is a characteristic feature of the Warburg effect and is crucial for the survival of cancer cells (9). Pyruvate kinase (PK) is a key rate-limiting enzyme for glycolysis that catalyzes the phosphorylation of phosphoenolpyruvate (PEP) and adenosine diphosphate (ADP) to produce pyruvate and adenosine triphosphate (ATP), which play critical roles in glycolysis during tumor formation (10). Since PKs are found in various tissues and exhibit distinct catalytic activities, it suggests that there may be different isotypes of this enzyme (11). There are four different subtypes of PKs that are expressed in specific tissues: muscle (M1), liver (L), erythrocyte (R), and ubiquitous (M2) (12). Among these subtypes, PKM2 is frequently overexpressed in cancer cells and has been extensively studied as a subtype specific to tumors (13).

Since Christofk et al. first demonstrated the necessity of PKM2 expression for cancer-specific aerobic glycolysis, known as the Warburg effect, there has been significant interest in its role in cancer development (14). In addition to its role in tumor metabolism, PKM2 plays a role in oncogenic cytokinesis, tumor growth, and metastasis (15–17). Furthermore, PKM2 functions as a protein kinase by phosphorylating its substrates and regulating gene expression (18). Previous studies have highlighted the importance of PKM2 in promoting cancer cell growth and survival (16, 19). Therefore, comprehending the biochemical functions of PKM2 during tumor progression is essential for identifying possible therapeutic targets and developing novel therapies for BC (5). Hence, this review highlights current advancements in understanding how PKM2 interacts with different transcription factors and proteins that influence the initiation and progression of BC. Additionally, it explored the impact of natural products and noncoding RNAs on various biological functions of BC cells by controlling the nonmetabolic functions of PKM2.

### Structure and function of PKM2

**Figure 1:**
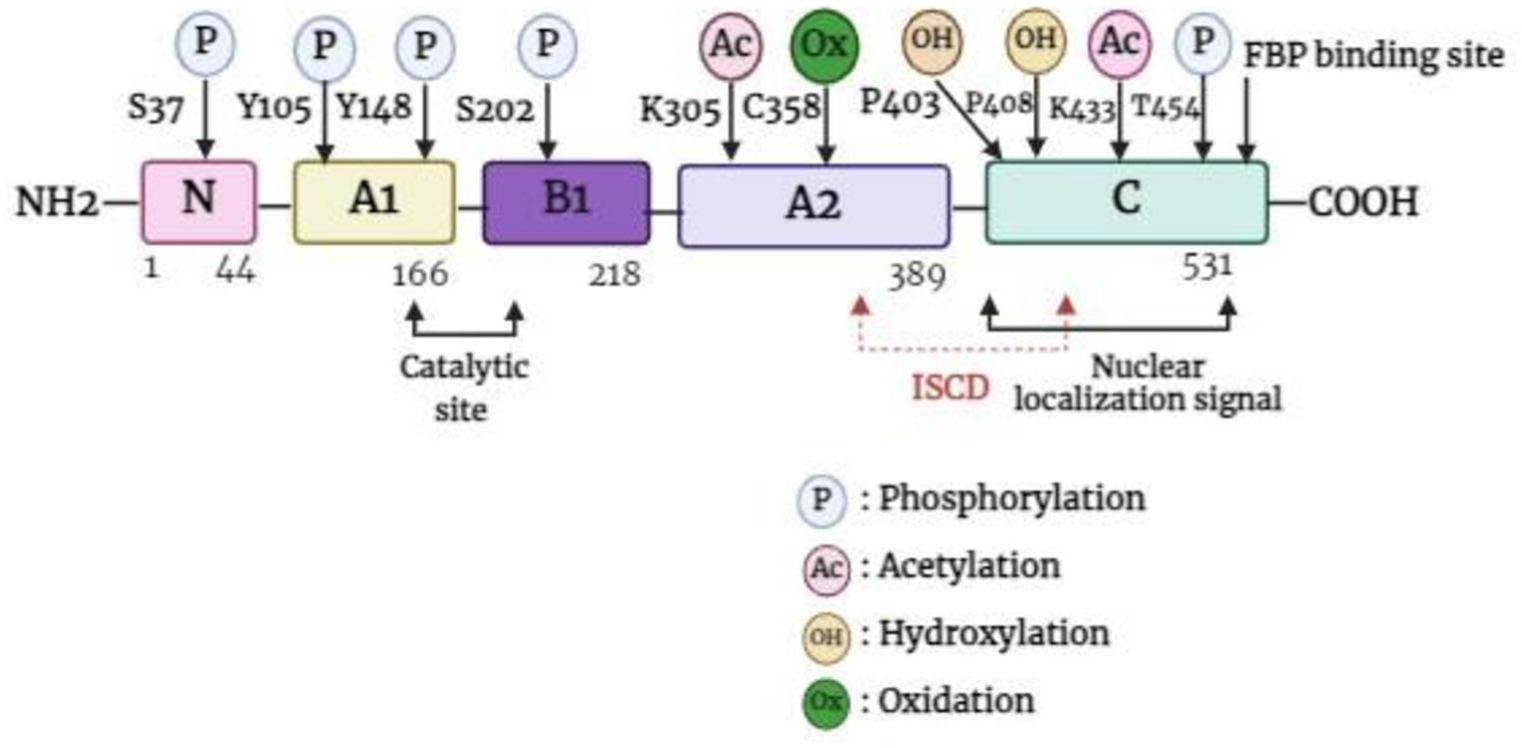
Basic structure of PKM2 monomer. FBP, fructose 1,6-bisphosphate; ISCD, inter-subunit contact domain.

The PKM gene, which is located in the 15q23 region of the chromosome, has the ability to undergo alternative splicing to produce either PKM1 or PKM2. The PKM gene spans approximately 32 kb and comprises 12 exons and 11 introns. The lengths of exons 9 and 10 are identical, which contributes to the differences in the final transcript. For PKM2, the final messenger ribonucleic acid (mRNA) product includes exon 10 but omits exon 9, a feature unique to PKM1 (20, 21). PKM2 is composed of 531 amino acids and has four domains: A (244 aa), B (102 aa), C (142 aa), and N (43 aa)-terminal domains (22). The catalytic active site is formed by the interface between the A- and B-domains, whereas the C-domain includes the fructose 1,6-bisphosphate (FBP) binding site, which acts as an allosteric activator. Additionally, the C-domain contains a nuclear localization signal sequence (NLS) and an inter-subunit contact domain (ISCD) (**Figure 1**) (22, 23). Furthermore, the arrangement of the C-domain plays a crucial role in explaining the differences observed in allosteric regulation by FBP among various PKM isoforms (22). The ISCD domain of PKM2 has a protein sequence that differs from that of its alternate splice variant, PKM1, by 23 amino acids, resulting in distinct properties such as allosteric regulation by FBP. These differences also enable PKM2 to interact with specific protein partners, including phosphotyrosine proteins (24).

**Figure 2:**
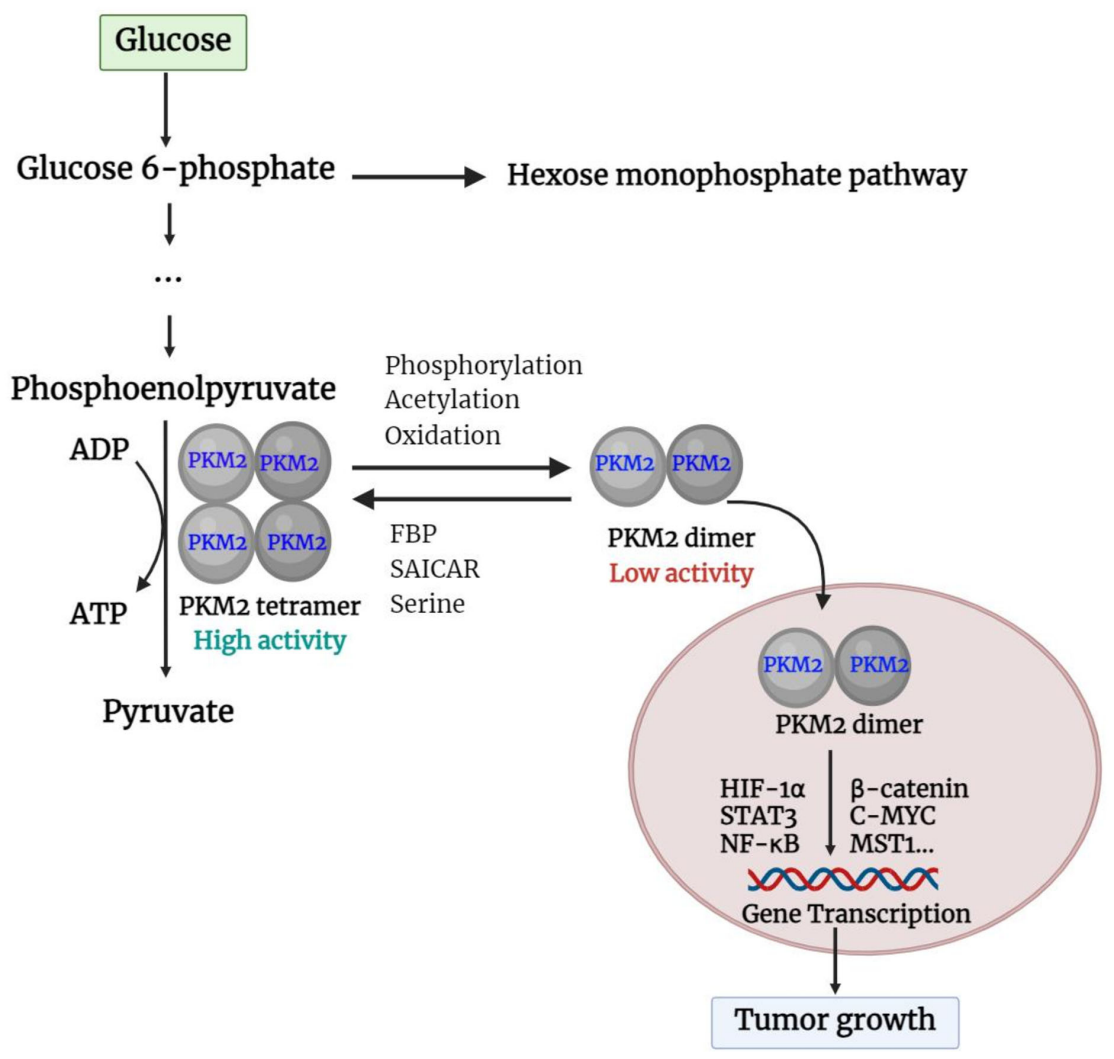
Basic function of PKM2 dimer and tetramer. PKM2, pyruvate kinase M2; FBP, fructose 1,6-bisphosphate; SAICAR, succinyl-5-aminoimidazole-4-carboxamide-1-ribose 5’-Phosphate; HIF-1α, hypoxia-inducible factor-1α; STAT3, Signal transducer and activator of transcription 3; NF-κB, nuclear factor kappa-light-chain enhancer of activated B cells; c-Myc, cellular-myelocytomatosis oncogene; MST1, mammalian sterile 20-like kinase 1.

Unlike other PK isoforms, such as PKL, PKR, and PKM1 which are exclusively tetramers, PKM2 is present in both tetrameric and dimeric forms. The A-domain of individual PKM2 units combines to form a dimer, and two such dimers bind at the interface of the ISCD (the C-domain) to create the full PKM2 tetramer (25). The shift between PKM2 dimers and tetramers is controlled by the structural changes caused by endogenous and exogenous activators and inhibitors (26). Fructose-1,6-bisphosphate (FBP) and serine are both potent allosteric activators of PKM2, which directly bind to PKM2 and stabilize it in the active tetramer configuration (27). In addition, when succinyl-5-aminoimidazole-4-carboxamide-1-ribose 5’-phosphate (SAICAR) binds to PKM2, it can trigger both the pyruvate kinase and the protein kinase activity of PKM2 (28). Furthermore, modifications such as phosphorylation, acetylation, and oxidation of PKM2 at the Tyr-105, Lys-305, and Cys-358 sites can prevent FBP from binding to tetrameric PKM2, thereby maintaining it in dimer form (29). PKM2 in tetrameric form is highly active at physiological concentrations of PEP and has a high affinity for PEP (30). In cases where PKM2 exists mainly in its highly active tetrameric form, as is the case in differentiated tissues and most normal proliferating cells, glucose is converted to pyruvate to produce energy (31). Meanwhile, PKM2 dimer is described by weak attraction to its substrate, PEP, and is virtually inactive at normal concentrations of PEP. In this form, PKM2 generates minimal ATP during the conversion of PEP to pyruvate, resulting in no net production of ATP through glycolysis (31, 32). This scenario occurs primarily in tumor cells, where PKM2 predominantly exists in the less active dimeric state. As a consequence, all glycolytic intermediates beyond PK accumulate and are redirected toward various synthetic processes such as nucleic acid production, phospholipid synthesis, and amino acid synthesis (26, 30, 33). Like tumor cells, cells that undergo rapid proliferation require a large amount of essential components such as nucleic acids, phospholipids, and amino acids (34). Importantly, the dimeric form of PKM2 can enter the nucleus and act as a protein kinase (35). The basic functions of the PKM2 dimer and tetramer are described in **Figure 2**.

### The effect of PKM2 on breast cancer tumorigenesis and development

**Figure 3:**
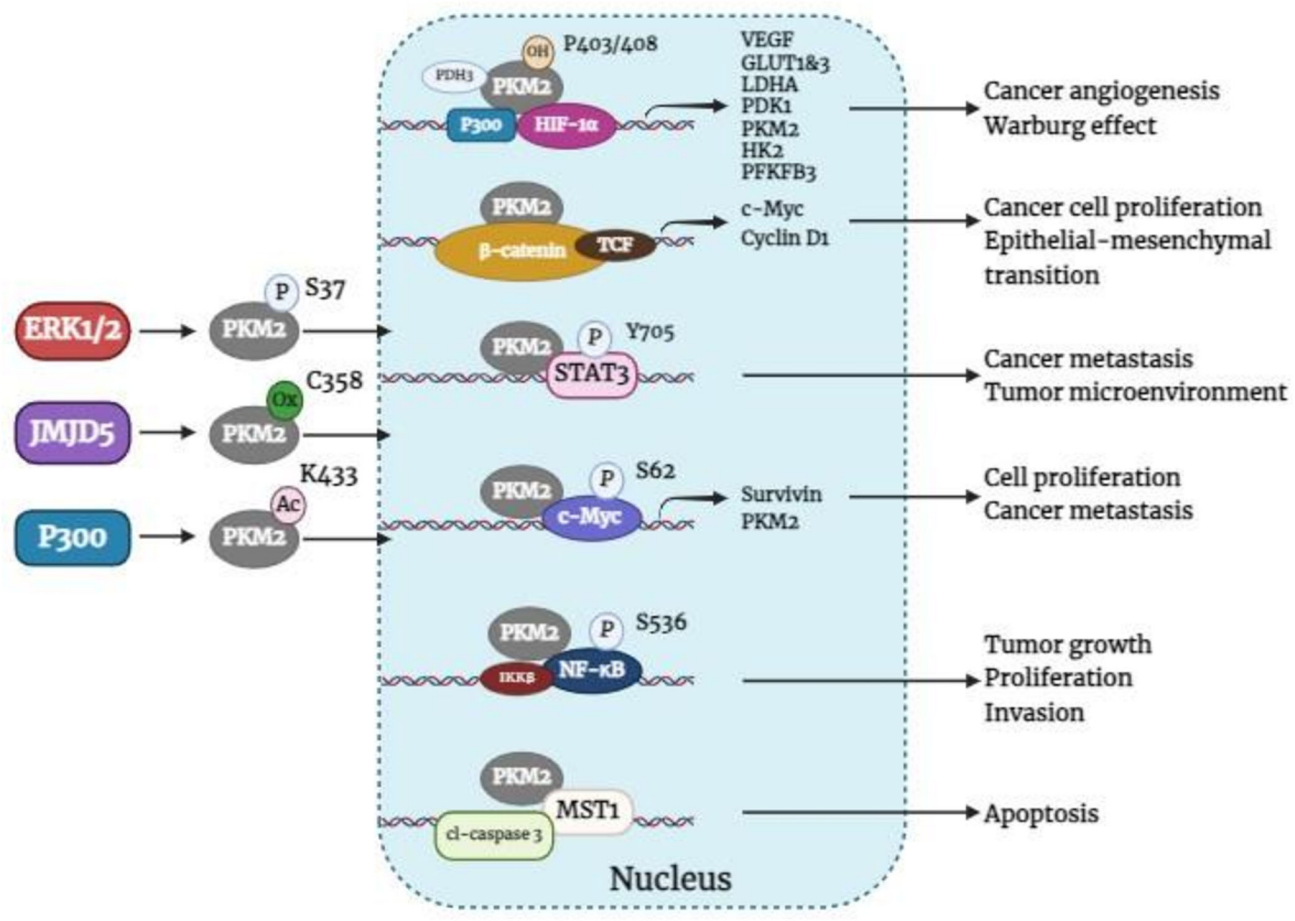
Nuclear PKM2 regulates the expression of related genes affecting BC tumorigenesis and development. ERK1/2, extracellular signal-regulated kinase 1/2; JMJD5, jumonji C domain-containing protein 5; PKM2, pyruvate kinase M2; HIF-1α, hypoxia-inducible factor-1α; PDH3, prolyl hydroxylase domain 3; TCF, T-cell factor; VEGF, vascular endothelial growth factor; GLUT1&3, glucose transporters 1 and 3; LDHA, lactate dehydrogenase A; PDK1, pyruvate dehydrogenase kinase 1, HK2, hexokinase 2; PFKFB3, 6-phosphofructo-2-kinase/fructose-2, 6-bisphosphatase 3; c-Myc, cellular-myelocytomatosis oncogene; MST1, mammalian sterile 20-like kinase 1; cl-caspase 3, cleaved-Caspase-3.

A large body of evidence supports the notion that cancers predominantly express PKM2 (14). Immunohistochemical analysis revealed that PKM2 is commonly expressed in BC (5). In BC, the activation of HIF-1α and epidermal growth factor receptor (EGFR) can facilitate the nuclear translocation of PKM2 (36), which is determined by the nuclear localization signal at its C-terminus. The phosphorylation of the PKM2 S37 site by extracellular regulatory protein kinases leads to its transformation from a tetramer to a monomer through the peptidyl-prolyl cis-trans isomerase NIMA-interacting 1 (PIN1), enabling the nuclear localization signal to enter the nucleus (23). In addition, acetylation of PKM2 at Lys433 decreases FBP binding to PKM2 and the conversion of monomers or dimers to tetramers but increases PKM2 nuclear import and protein kinase activity (37). Furthermore, the Jumonji C domain-containing dioxygenase Jumonji domain-containing protein 5 (JMJD5) interacts directly with PKM2, promoting its movement into the nucleus and HIF-1α-mediated transactivation. The interaction between JMJD5 and PKM2 occurs at the intersubunit interface region of PKM2, preventing its tetramerization and inhibiting its kinase activity (38). Once within the nucleus, PKM2 serves as a transcriptional co-activator and stimulates the activation of various transcription factors, such as HIF-1α, β-catenin, STAT3, C-MYC, NF-κB, etc., which influences the expression of their respective downstream target genes. Overall, **Figure 3** shows how nuclear PKM2 regulates gene expression in relation to breast cancer development and progression.

#### PKM2/HIF-1α

Cancer cells exhibit unregulated cell proliferation, unrestricted cell division, and suppression of autophagy, making them reliant on additional oxygen and nutrients for survival (39). To meet these demands, tumor cells can utilize angiogenesis and metabolic alterations. The transcription factor HIF-1α plays a crucial role in regulating genes related to angiogenesis and the Warburg effect, both of which contribute to tumor formation (40). Previous studies have focused primarily on HIF-1α as a transcription factor that increases the expression of various glycolysis-related genes, such as glucose transporters (GLUT-1 and GLUT3) and enzymes involved in glycolytic pathways [lactate dehydrogenase A, pyruvate dehydrogenase kinase 1, hexokinase 2 (HK2), and PKM2], to promote BC glycolysis and consequently affect the proliferation of BC cells (36). Intriguingly, studies have revealed that PKM2 can influence the expression and activation of HIF-1α (41, 42). The activation of PKM2 in hypoxic BC cells leads to the activation of NF-κB/p65 and HIF-1α, resulting in increased production and secretion of vascular endothelial growth factor (VEGF), which promotes angiogenesis and tumor growth (41). Angiogenesis plays a crucial role in tumor invasion and migration. Notably, studies have shown that vascular endothelial growth factor A (VEGFA) can enhance the self-renewal of cancer stem cells and promote epithelial–mesenchymal transition (EMT), potentially contributing to tumor metastasis in BC cells (43). Furthermore, clinical studies have shown that patients with metastatic BC have increased circulating VEGFA levels (44, 45). Consequently, targeting VEGF through the PKM2/HIF-1α axis in BC is a feasible approach. Chai et al. (46) reported that silencing PKM2 in BC cells resulted in reduced expression of HIF-1α and VEGF, as well as cell migration capabilities. These findings suggest that PKM2 influences the expression of HIF-1α and subsequently affects the onset and progression of various tumors, including BC. Specifically, prolyl hydroxylase domain 3 (PDH3) hydroxylates PKM2 at proline 403/408, leading to its binding to the HIF-1α subunit, which enhances HIF-1α binding to p300. This interaction then recruits p300 to the hypoxia response element, facilitating the transactivation of HIF-1α target genes and promoting PKM2 transcription (47). PKM2 and HIF-1α establish a positive feedback loop. Recent investigations have revealed that in BC cells under hypoxic conditions, nuclear PKM2 recruits HIF-1α and p300, leading to the upregulation of 6-phosphofructo-2-kinase/fructose-2,6-bisphosphatase 3 (PFKFB3), an enzyme involved in glycolysis that is associated with cancer progression and aggressiveness (36). These findings suggest that nuclear PKM2 can induce glycolysis by activating HIF-1α, potentially influencing various biological processes in BC. These results underscore the potential of targeting the PKM2/HIF-1α axis as a promising strategy for anti-BC therapies.

#### PKM2/β-catenin

β-catenin is the central component of the Wnt signaling pathway and plays a key role in the regulation of cell proliferation, differentiation, and apoptosis (48). The Wnt-signaling pathway is an evolutionarily conserved and complex signaling cascade that plays crucial roles in both development and disease (49). The Wnt signaling pathway plays an important role not only in the development and maintenance of healthy breast and mammary glands but also in BC etiology (50). To validate the regulation of β-catenin by PKM2, Zhao and colleagues investigated the levels of β-catenin in MDA-A-231-shPKM2 and BT20-PKM2 cells. The findings demonstrated that β-catenin expression was suppressed in PKM2-silenced cells, whereas both the protein and RNA levels of β-catenin were increased in PKM2-overexpressing cells (51). In addition, several studies have indicated that PKM2 is translocated into the nucleus in cancer cells, where it acts as a transcription factor and controls β-catenin transactivation (52, 53). Once inside the nucleus, PKM2 can interact with the transcription factor T-cell factor (TCF), leading to an increase in the transcription of the c-Myc and cyclin D1 genes. This process ultimately facilitates BC cell proliferation and EMT (51, 54). Notably, the involvement of β-catenin in various cancer cell activities, such as cell migration, invasion, and angiogenesis, is noteworthy. Findings from a clinical investigation indicated a potential correlation between the expression of β-catenin in TNBC tissue and survival prognosis (52). A recent study revealed that cryptotanshinone significantly reduced BC migration and invasion, possibly through the suppression of the PKM2/β-catenin pathway and the overexpression of PKM2, which decreased cryptotanshinone sensitivity in BC cells (54). Consequently, these findings suggest that targeting the PKM2/β-catenin signaling pathway may hold promise for treating BC.

#### PKM2/STAT3

STAT3 is activated in several types of tumor cells and can promote the malignant transformation of cells and inhibit apoptosis, suggesting that the STAT3 signaling pathway could be a new target for tumor gene therapy (55, 56). The Janus kinase (JAK)/STAT3 pathway is responsible for regulating the gene expression of several enzymes involved in glucose metabolism. Recently, interest in understanding the importance of the PKM2/STAT3 pathway in the advancement of cancer has increased (57). PKM2 overexpression facilitates the nuclear translocation of STAT3, a transcription factor crucial for PKM2-driven metastasis. The protein kinase activity of PKM2 mediates the nuclear translocation and up-regulation of STAT3, which regulates the aggressive progression of colorectal cancer (58). Nuclear PKM2 activates the transcription of MAP kinase kinase 5 (MEK5/ERK5) by phosphorylating STAT3 at Y705, resulting in cell proliferation (18, 59). Some investigations related to the STAT3 oncogene have laid the foundation for the interpretation and treatment of BC formation mechanisms (60). Guan et al. reported that knocking down PKM2 in BC cells resulted in a decrease in the expression of STAT3 and STAT3 (Tyr(P)-705), leading to the suppression of gene transcription and the inhibition of BC cell proliferation (61). *In vitro* and *in vivo* studies have confirmed that pY705 modification of STAT3 is necessary for tumor growth, autophagy, and metastasis and suggest that STAT3 is an effective approach for treating cancer (62). Furthermore, clinical studies using STAT3 inhibitors indicate encouraging outcomes in malignant conditions. For example, yuanhuacine (YHC), a daphnane-type diterpenoid as the main active ingredient, may inhibit BC cell proliferation and induce apoptosis *in vivo* and *in vitro* by regulating the STAT3 pathway and glycolysis through targeting PKM2 (63). The suppression of STAT3 leads to apoptosis, inhibition of growth, reduced tumor cell invasion, and increased sensitivity to treatment in BC cells (64). These results emphasize the potential significance of targeting PKM2/STAT3 as a therapeutic approach for BC.

#### PKM2/C-MYC

c-Myc is a frequently activated oncogene that is closely associated with the initiation and progression of cancer in humans (65). It functions as an oncoprotein involved in various cellular processes such as deoxyribonucleic acid (DNA) replication, transcription, and RNA splicing (66). One crucial transcriptional target of c-Myc is survivin, which is an inhibitor of the apoptosis protein family and plays a significant role in tumorigenesis (67–69). Survivin is highly expressed in BC and promotes the proliferation and migration of cancer cells (67, 70). Transcriptional factors play a role in the development and advancement of cancer, such as metastasis and cellular proliferation (71). PKM2’s nuclear translocation enables it to serve as a transcriptional activator for the c-Myc gene (70, 72, 73). Yu et al. (70) revealed that PKM2 interacts with c-Myc and regulates its phosphorylation at Ser-62. This interaction suggests that c-Myc may be a novel substrate of PKM2. Inhibiting PKM2 reduces c-Myc phosphorylation, leading to the downregulation of c-Myc protein expression through the promotion of its degradation in BC cells. The results of c-Myc knockdown indicated decreased survivin protein and mRNA levels, suggesting that PKM2 regulates survivin via c-Myc (70, 74). Thus, a novel approach to inhibit BC cell proliferation and migration could involve focusing on the PKM2-c-Myc-survivin pathway.

#### PKM2/NF-κB

The nuclear factor kappa-light-chain enhancer of activated B cells (NF-κB) family of transcription factors plays important roles in regulating inflammation, immunological response, cell differentiation, proliferation, and survival (75). NF-κB consists of five subunits: p65/RelA, c-Rel, RelB, p50/NF-κB1, and p52/NF-κB2, which form unique protein complexes that bind to consensus DNA sequences at gene promoter regions to control various biological activities (76, 77). PKM2 plays a role in controlling the NF-κB signaling pathway in cancer cells. For example, Azoitei et al. reported that PKM2 stimulated the release of VEGF-A by activating NF-κB and HIF-1α, which in turn affected tumor angiogenesis in pancreatic cancer (41). In addition, Zheng et al. found that PKM2 facilitates the movement of NF-κB/P65 to the nucleus, resulting in the promotion of ovarian cancer cell migration and invasion (78). In BC, there has also been evidence of positive crosstalk between NF-κB and PKM2 (79, 80). Knockdown of PKM2 in TNBC cells significantly reduces the activity of NF-κB by decreasing the phosphorylation of p65 at serine 536 and suppressing the expression of NF-κB target genes (80, 81). However, the exact mechanism by which PKM regulates the activity of NF-κB through p65 phosphorylation remains unclear. IKKβ is known to play a critical role in phosphorylating RelA/p65 at serine 536 and controlling the activation of NF-κB (81). Dimeric PKM2 acts as an active protein kinase that can phosphorylate certain nuclear proteins (18, 82). Therefore, future investigations will explore whether the localization of PKM2 differs between the cytoplasm and nucleus and whether dimeric PKM2 can phosphorylate the p65 protein at serine 536 or influence IKKβ signaling molecules. These studies may reveal additional mechanisms and metabolic changes in PKM2/NF-κB in TNBC cells.

#### PKM2/MST1

Mammalian sterile 20-like kinase 1 (MST1), also known as STK4 and Krs-2, is widely present in all human tissues and cell lines and serves as a crucial upstream kinase in the Hippo signaling pathway (83). MST1 plays a vital role in regulating mammalian cell shape, controlling organ size, managing apoptotic responses, and influencing cancer development (83, 84). PKM2 physically interacts with MST1 *in vivo*. The phosphorylation and proteolysis of MST1 regulated by PKM2 could affect downstream signaling pathways controlled by MST1, which are involved in regulating cell death (85, 86). Treatment with 4-hydroxytamoxifen reduces the levels of PKM2 and disrupts its interaction with MST1, leading to the activation of caspase-3. This further enhances the cleavage of MST1 mediated by caspase-3 in BC cells. The cleaved form of MST1 relocates to the nucleus, where it causes chromatin condensation and ultimately triggers cell apoptosis (85). Silencing PKM2 can activate Caspase-3, which then cleaves its substrate to enhance the apoptotic signaling cascade induced by tamoxifen treatment (85, 87). These findings offer new insight for the development of therapies against BC.

### Other transcription factors or signaling pathways affected by nuclear PKM2

Besides, studies have revealed that the activation of PKM2 induces the phosphorylation of adenosine monophosphate-activated protein kinase (AMPK), resulting in the phosphorylation and subsequent inhibition of acetyl-CoA carboxylase. This suggests that heightened PK activity results in an energy deficit (88). AMPK activation is known to hinder mammalian target of rapamycin (mTOR) signaling during low-energy conditions, exerting a cytostatic effect (89). The classic antidiabetic drug metformin, for example, has been extensively studied for its anticancer effects via AMPK signaling (90, 91). Existing studies have demonstrated that PKM2 interacts directly with the histone methyltransferase enhancer of zeste homolog 2 (EZH2) to orchestrate the epigenetic silencing of the carnitine transporter, SLC16A9. Inhibiting PKM2 disrupts the binding of EZH2 to SLC16A9, which de-represses the expression of SLC16A9 and increases intracellular carnitine influx, thus programming TNBC cells toward fatty acid oxidation-dependent and luminal-like cell states (92). According to previous reports, nuclear PKM2 promotes the proliferation of liver, breast, colon, and lung cancer cells by phosphorylating nuclear sterol regulatory element-binding protein (SREBP)-1a T59, which prevents SREBP-1a from being ubiquitinated and degraded and consequently enhances the expression of SREBP-1a’s target genes related to lipid metabolism. This study demonstrated that PKM2 functions as a transcriptional co-activator by promoting SREBP-1a’s transcriptional activation function and thus driving the proliferation of BC cells (93).

### The effect of regulating nonmetabolic enzyme function of PKM2 on breast cancer tumorigenesis and development

The above studies revealed that the nuclear localization of dimeric PKM2 can affect a variety of biological processes in BC cells. As a result, controlling the nonmetabolic function of PKM2 is critical for intervening in BC carcinogenesis and progression. Currently, a growing body of research is focused on exploring the potential role of natural products and noncoding RNAs in regulating the nonmetabolic function of PKM2 to affect BC tumorigenesis and development.

### Natural products regulate the nonmetabolic function of PKM2

#### Shikonin

Shikonin, an active compound extracted from *Lithospermum erythrorhizon*, has exhibited various pharmacological effects and has been found to possess antitumor capabilities in a variety of human cancer types (94, 95). Specifically, it can impede the glycolytic function of PKM2 in BC cells (96, 97), and suppress its nonmetabolic enzyme function to impact the biological processes of these cells (36, 97–99). Shikonin works by binding with the R399/400 residues of PKM2 to hinder its nuclear translocation, hence leading to the disruption of the PKM2-HIFs-PFKFB3 pathway (36). As PFKFB3 facilitates aerobic glycolysis, reducing its levels increases the reliance on OXPHOS to meet ATP needs (100). Cancer cell growth is known to be inhibited by a transition from a glycolysis-dependent state to an OXPHOS-dependent state (101). Pandkar et al. found that shikonin inhibits the hypoxic activation of PFKFB3 by engaging with a putative nuclear localization signal (NLS) on PKM2, thereby blocking its movement into the nucleus during hypoxia. Furthermore, studies in mice carrying WT BBS MCF7 tumors revealed that shikonin treatment led to significantly reduced tumor growth, emphasizing the crucial role of targeting PKM2 signaling to inhibit the progression of tumors (36). Moreover, LEE et al. showed that shikonin is a robust and rapid inducer of late apoptosis in MDA-MB-231 cells, implying that inhibitors of PKM2 may be validated treatment targets for TNBC. Targeting PKM2 in TNBC paves the way for the development of PKM2 inhibitors as potential anti-TNBC agents (98). Therefore, focusing on targeting PKM2 in BC sets the stage for developing promising anti-BC agents in the form of PKM2 inhibitors.

#### Cryptanshinone

Cryptotanshinone is a liposoluble monomer of tanshinones isolated from the dried roots and rhizomes of *Salvia miltiorrhiza* (102). Cryptotanshinone has been recognized as a medication with anti-inflammatory and anti-oxidative properties, and it has demonstrated its ability to combat different forms of cancer by impeding cell growth, movement, and infiltration (103). Notably, significant evidence suggests that cryptotanshinone has the capacity to inhibit the metabolism of glucose in ovarian cancer cells, suggesting that it could be an effective anti-cancer agent by controlling glycolysis (104). Zhou et al. showed that cryptotanshinone has an anti-cancer effect on BC cells by inhibiting cell proliferation, migration, and invasion *in vitro*. In addition, it significantly decreases the expression of glycolysis-related proteins and PKM2/β-catenin signaling in BC cells (54). PKM2 is a crucial regulator that controls the transactivation of β-catenin, which is responsible for numerous critical functions of cancer cells, including invasion, migration, and angiogenesis (105). Cryptotanshinone successfully hindered the migration and invasion of BC, which could be attributed to the suppression of the PKM2/β-catenin pathway (54). Therefore, cryptotanshinone may hold promise for the advancement of novel targeted medications for BC.

#### Cyclosporine A

Cyclosporin A is an immunosuppressant that is not toxic to cells and was first identified in the 1970s. Initially, it was utilized to suppress immune responses after organ and marrow transplantation (106). Afterwards, it has been utilized in various medical fields where autoimmune or inflammatory processes are involved in pathology (107, 108). Recent studies have highlighted the potential antitumor activity of this compound against different cancer cells, including BC. Jiang et al. found that cyclosporin A inhibited the cell proliferation, cell cycle progression, and G1/S phase transition of BC cell lines (109). Several studies have reported that cyclosporin A hinders the expression of some oncogenes. Cyclosporin A can inhibit the proliferation of cancer cells, possibly by controlling the expression levels of c-Myc, p21, and proliferating cell nuclear antigen (PCNA) through the suppression of calcineurin (CaN)/nuclear factor of activated T cells (NFAT) activity (110). It also suppresses cancer-specific PKM2, which supports the advancement of the tumor (109, 111). The function of the PKM2 enzyme was also suppressed in MCF-7 cells when they were exposed to cyclosporin A. Pyruvate kinase catalyzes the final step in the glycolytic pathway and is responsible for net ATP production; hence, the production of ATP was evaluated in MCF-7 cells treated with cyclosporin A and was found to be significantly reduced (109). This finding shows that cyclosporin A hampers the expression of PKM2, leading to a reduction in intracellular ATP within tumor cells, which ultimately slows down cell growth and may even trigger cell death (109, 111). Thus, cyclosporin A acts as a suppressor of BC growth by targeting tumor-related PKM2.

#### Lapatinib

Lapatinib is a small-molecule tyrosine kinase inhibitor that targets EGFR and HER2 (112, 113). Clinical studies have revealed that lapatinib is well-tolerated and can be used alone or in combination with other drugs for the treatment of BC (114, 115). The specific molecular mechanism of lapatinib involves regulating PKM2-mediated STAT3 tyrosine phosphorylation (18, 61). Guan et al. (61) demonstrated that lapatinib hinders the proliferation of BC cells by influencing the expression of PKM2, which decreases the levels of STAT3 and phosphorylated STAT3. Downregulation of PKM2 expression by lapatinib-mediated EGFR and HER2 suppression decreases STAT3 and phosphorylated STAT3 expression, resulting in decreased gene transcription and prevention of tumor cell proliferation.

#### Mangifera indica

M. indica, also known as mango, is a member of the *Anacardiaceae* flowering family (116). Various sections of the M. indica plant contain different types of phytochemicals, and they have traditionally been used to treat a wide range of health issues such as gastrointestinal, genitourinary, ophthalmic, and respiratory conditions (117, 118). Preclinical research on extracts derived from diverse plant sections has indicated their anti-cancer, anti-inflammatory, antimicrobial, antioxidant, and immunomodulatory properties (119, 120). Specifically, various investigations have reported the anti-cancer properties of the pulp extracts of M. indica in BC. These findings suggest that consuming M. indica fruits could be beneficial for BC treatment (121). M. indica extracts (leaf, bark, and seed coats) were also found to have anticancer activity against TNBC (122, 123). These studies revealed that M. indica extracts could inhibit PKM2, but the exact mechanism underlying these effects is still not fully understood. Further research is also suggested to examine their potential in *in vivo* studies.

#### Carpesium abrotanoides (L.)

Carpesium abrotanoides Linn. (CA) is a popular medicinal plant recognized for its anti-inflammatory effects and wide range of therapeutic applications (46). Some studies have demonstrated that CA extracts exhibit promising antitumor effects on various cancer cells *in vitro*, including non–small cell lung, ovarian, skin, and colon cancer cells (124, 125). As a traditional herbal remedy, it is commonly used as an oral decoction for treating chronic inflammatory illnesses (126). Currently, various compounds have been isolated from the whole plant of CA that display significant cytotoxicity against MCF-7 and MDAMB-231 cells, indicating its potential anti-BC effects (46, 127). In addition, Chai et al. found that CA has dose-dependent antiproliferative effects on both metastatic (MDA-MB-231) and nonmetastatic (MCF-7) BC cells while inhibiting their migration ability. Furthermore, CA downregulates the expression of glycolysis-related proteins (PKM2, LDHA, HK2, and GLUT1) and suppresses the PKM2/HIF-1α/VEGF signaling pathway to exert its anticancer effects (46). In cancer cells, PKM2 activates the transcription of HIF-1α and its target gene VEGF in the nucleus; this stimulates the secretion of VEGF to promote angiogenesis and enhance tumor growth (41). Angiogenesis is a key event in tumor invasion and migration. Moreover, VEGFA has been linked to promoting cancer stem cell self-renewal and EMT while contributing to tumor metastasis in BC cells (43). Clinical studies have also revealed that patients with metastatic BC tend to have higher circulating levels of VEGFA (45). Consequently, targeting the PKM2/HIF-1α axis holds promise as a feasible approach for regulating VEGF in BC.

#### Yuanhuacine

Yuanhuacine, a daphnane-type diterpenoid as the main active ingredient, has been previously documented to exhibit cytotoxic effects *in vitro* against a wide range of human cancer cell lines (128–130). For instance, yuanhuacine has notable inhibitory effects on human lung cancer cells (131). Recent studies have revealed that yuanhuacine significantly inhibits effect BC cell growth (63, 132). Yuanhuacine reduced the growth of BC cells and caused apoptosis both *in vivo* and *in vitro*. The mechanism of action has been linked to yuanhuacine, which has been hypothesized to potentially disrupt the interactions of PKM2 and STAT3, hence inhibiting downstream proteins. Besides, yuanhuacine suppresses BC cells by targeting PKM2, which controls the STAT3 pathway and glycolysis. The combination of yuanhuacine and PKM2 siRNA treatment significantly hindered the phosphorylation of STAT3 (Y705) and its downstream effects, in contrast to yuanhuacine treatment alone (63). This finding could also indicate a synergistic effect of yuanhuacine when it is used with other cancer-specific treatments.

#### Cantharidin

Cantharidin, a sesquiterpenoid bioactive component, is among the active ingredients found in *mylabris.* Many *in vitro* studies have investigated cantharidin’s antitumor activity, which includes hindering migration, inducing apoptosis, halting the G2/M transition, and suppressing invasion (133). Yang et al. showed that EGFR activation results in the translocation of PKM2 into the nucleus, leading to increased expression of GLUT-1 and LDHA, which work together to enhance aerobic glycolysis in a positive feedback loop (105). This mechanism relies on ERK signaling, which is facilitated by importin α5 (23). Pan et al. (134) investigated the mechanism by which cantharidin influences PKM2 translocation into the nucleus in BC cells. Cantharidin reduces GLUT1 transcription and glucose uptake by blocking the transformation of PKM2 dimers into tetramers and their entry into the nucleus. Most importantly, the inhibitory effect of cantharidin on migration and invasion was significantly reversed when FBP and L-cysteine were introduced (134). These findings suggest that cantharidin and its derivatives have strong potential as anti-metastatic agents, making them highly valuable in clinical applications.

#### Beta‐elemene

Beta-elemene is a prominent non‐cytotoxic anticancer ingredient extracted from *Curcuma zedoary* that triggers cancer cell death, halts cell cycle progression, and improves radiotherapy and chemotherapy sensitivity without causing myelosuppression or notable harm to the liver or kidney (135–137). In cancer cells, the balance between tetrameric and dimeric forms of PKM2 regulates the glucose metabolic pathway, influencing energy production and the synthesis of anabolic precursors. Additionally, this interconversion between dimeric and tetrameric PKM2 helps to maintain a dynamic equilibrium (37, 138). Pan et al. showed that β‐elemene inhibited the spread of BC by impeding aerobic glycolysis. Specifically, β‐elemene hindered the transformation of dimeric and tetrameric forms of PKM2, thus inhibiting its pyruvate kinase activity, resulting in reduced utilization of glucose and generation of pyruvate and lactate. Besides its metabolic function, PKM2 acts as a nonmetabolic protein kinase and transcriptional coactivator for HIF-1α and c-MYC, which are crucial for tumorigenesis induced by the activation of EGFR. Activated EGFR facilitates the translocation of PKM2 to the nucleus through importin α5 (23, 105). β‐elemene inhibited the EGFR‐importin α5‐mediated movement of PKM2 into the nucleus and the expression of GLUT1, monocarboxylate transporter 1 (MCT1), MCT4, and LDHA (139). Collectively, these studies suggest that β‐elemene can inhibit BC metastasis by disrupting PKM2‐mediated metabolic signaling to exert an anti-BC effect.

### Noncoding RNAs regulate nonmetabolic function of PKM2 in breast cancer

Current studies are focused on the impact of long noncoding RNA (lncRNA), circular RNA (circRNA), and microRNA (miRNA) on PKM2 in BC tumorigenesis and development (140–142). Noncoding RNA can influence BC cell metabolic reprogramming by targeting PKM2’s metabolic enzyme function as well as its nonmetabolic enzyme function (141, 143). In some cases, miRNAs have been found to regulate PKM2 expression in BC cells. For instance, miR-122 is abundantly released by tumor cells and promotes metastasis by adapting to the metabolic environment in the pre-metastatic niche, whereas down-regulation of PKM2 and GLUT1 limits glucose consumption in BC cells (144). miR-Let-7a inhibited MDA-MB-231 cell proliferation and particularly upregulated the levels of PMK2, oxidative phosphorylation, and reactive oxygen species (ROS) in TNBC and improved the sensitivity of BC cells to the tumor suppressor doxorubicin in BC metastasis (145). CircKIF4A, which is largely found in the cytoplasm, may interact with miR-335, while aldolase A (ALDOA) and octamer-binding transcription factor 4 (OCT4) serve as miR-335’s downstream targets. ALDOA and OCT4 are metabolic proteins that control glycolytic proteins such as HK2 and PKM2 (146–148). Increased miR-335 expression resulted in decreased levels of ALDOA/HK2 and OCT4/PKM2 proteins. As a result, circKIF4A regulates glucose metabolism through the miR-335-ALDOA/OCT4-HK2/PKM2 pathway (149).

Zheng et al. demonstrated the importance of HIF-1α antisense lncRNA (HIFAL) in maintaining and increasing HIF-1α-driven transactivation and glycolysis. Functionally, HIFAL recruits PHD3 to PKM2, causing its prolyl hydroxylation and facilitating the entry of the PKM2/PHD3 complex into the nucleus by interacting with heterogeneous nuclear ribonucleoprotein (hnRNP) F. This promotes HIF-1α transactivation, glucose absorption, and lactate generation in BC cells (150). Besides, Yao et al. revealed that miR-Let-7a-5p suppresses aerobic glycolysis by modulating the STAT3/hnRNP-A1/PKM2 signaling pathway. They showed that hnRNP-A1 controls PKM2 gene selective cleavage, resulting in enhanced PKM2 synthesis. STAT3 may increase hnRNP-A1 transcription, whereas miR-Let-7a-5p suppresses STAT3 and hence reduces PKM2 (141, 151).

A study conducted by Wen et al. revealed that miR-152 suppresses growth and angiogenesis in BC by suppressing both β-catenin and PKM2. β-catenin, which is a downstream target of insulin-like growth factor 1 (IGF-1), plays a role in controlling cell proliferation (53). Recent evidence has shown that miR-148a/152 activation contributes to the suppression of PKM2 and NF-κB p56 expression in TNBC cells. NF-κB p56 interacts with PKM2 to regulate early growth response 1 (EGR1) expression. EGR1 can bind to multiple sites on miRNA gene promoters, thereby controlling miR-148a and miR-152 expression (152).

## Conclusion and future perspectives

In this review, we reviewed how PKM2 has emerged as a crucial player in BC; through its role as a transcriptional co-activator and regulator of gene expression, PKM2 has been demonstrated to contribute to the epigenetic regulation of gene transcription. Understanding the diverse functions of PKM2 beyond its metabolic role opens new avenues for targeting this enzyme in BC treatment. The nonmetabolic role of PKM2 controls the expression of cancer-related genes implicated in various aspects of BC progression, such as cell cycle, proliferation, programmed cell death, angiogenesis, migration and invasion, tumor microenvironment, and others. Natural products such as shikonin, cryptanshinone, cyclosporine A, lapatinib, mangifera indica, beta-elemene, yuanhuacine, cantharidin, and others control PKM2’s nonmetabolic function and affect BC progression. In addition, the interaction of PKM2 with noncoding RNAs has garnered increasing interest. Noncoding RNAs regulate PKM2’s nonmetabolic enzyme function and influence BC growth. In the future, it will be imperative to further investigate the nonmetabolic function of PKM2 in BC in order to gain a comprehensive understanding of its involvement in the growth and advancement of tumors. In addition, exploring the interaction between metabolic reprogramming and PKM2-mediated nonmetabolic functions in BC is an intriguing area for further research. This knowledge could potentially lead to the use of PKM2 as a molecular marker for BC diagnosis and prognosis, which could be highly important for the therapeutic targeting of BC.
